# Application of geometric morphometrics for variety identification in *Rubus crataegifolius*: a comparison of primocane and floricane leaf morphology

**DOI:** 10.3389/fpls.2026.1799767

**Published:** 2026-07-14

**Authors:** Seol-Jong Kim, Yoon-Young Kim, Je Min Park, Soon-Ho Kwon

**Affiliations:** National Forest Seed Variety Center (NFSV), Chungju, Chungcheongbuk-do, Republic of Korea

**Keywords:** breeding, Elliptic Fourier descriptors, Korean raspberry, morphometrics, phenotypic analysis, *Rubus crataegifolius*, variety identification

## Abstract

Accurate identification of crop varieties is essential for plant breeding programs and the protection of Plant Breeders’ Rights (PBR), yet traditional morphological assessment methods remain subjective and time-consuming, particularly for species with complex morphological diversity such as *Rubus crataegifolius*. This study demonstrates that geometric morphometric techniques provide an objective, quantitative complementary approach for distinguishing Korean raspberry varieties, addressing the limitations of subjective visual assessment while remaining compatible with molecular marker analysis. We employed three complementary morphometric approaches: landmark-based analysis (19 anatomical points capturing vein junctions and leaf margins), Elliptic Fourier Descriptors (EFD) for outline contours, and a hybrid landmark-EFD dataset. Using these approaches, we analyzed primocane and floricane leaves from 10 accessions of *R. crataegifolius* comprising 8 varieties and 2 landraces and performed principal component analysis (PCA) and linear discriminant analysis (LDA) with leave-one-out cross-validation. As a result, among the three morphometric approaches applied to primocane and floricane leaves, landmark-based analysis of primocane leaves achieved the highest classification accuracy (87.2%), with an overall average accuracy of 72.0% (range: 51.1–87.2%) across all six analytical combinations. LDA visualization suggested the presence of four major morphological groups, and primocane leaves exhibited higher discriminatory power than floricane leaves, which may reflect greater morphological uniformity under normal growing conditions. Landmark analysis effectively detected subtle differences in leaf venation and leaflet architecture that are difficult to distinguish visually, highlighting the capacity of morphometrics for objective and multidimensional morphological analysis. These findings suggest that morphometric analysis provides a practical and cost-effective preliminary screening tool, complementary to molecular approaches, for supporting Distinctness, Uniformity, and Stability (DUS) examination in raspberry variety evaluation. This approach shows strong potential for offering a scalable solution for variety registration and protection and supporting sustainable horticultural development.

## 1 Introduction

The genus *Rubus* L. (Rosaceae), which includes raspberries (subgenus Idaeobatus), blackberries (subgenus *Rubus*), and their hybrids, represents one of the most economically and culturally significant horticultural crops worldwide (Gao et al., 2023). With over 800 species and numerous registered varieties (Huang et al., 2023), *Rubus* contributes substantially to global fruit production (Quideau, 2009; Hummer, 2010), valued for their nutritional benefits, including high levels of antioxidants, vitamins, and dietary fiber (Rocabado et al., 2008; Schulz and Chim, 2019; Bhatt et al., 2023). Among these, the Korean raspberry (*Rubus crataegifolius* Bunge) holds particular importance in East Asia. Native to Korea, Japan, northeastern China, and parts of far eastern Russia (Moon et al., 2011), this species has been utilized for many years in traditional medicine and as a food source due to its bioactive compounds, such as ellagic acid and flavonoids, which exhibit anti-inflammatory and anticancer properties (Choi et al., 2016). In Korea, *R. crataegifolius* is cultivated not only for its edible fruits but also for its potential in breeding programs aimed at enhancing disease resistance and yield (Hwang et al., 2021). It has also been used in interspecific crosses, contributing pest resistance traits such as wound periderm formation against cane midge (McNicol et al., 1983; Knight, 1993). However, the species’ wide morphological diversity, influenced by environmental factors and potential genetic hybridization with closely related taxa, poses significant challenges for accurate variety identification and classification (Cho et al., 2009; Yang et al., 2019; Gao et al., 2023).

Traditional methods for variety identification in *Rubus*, including visual morphological assessments and simple linear measurements (e.g., leaf length-to-width ratios or petiole length), have long been the standard in breeding and registration processes (UPOV, 2023). These approaches are integral to Distinctness, Uniformity, and Stability (DUS) tests mandated by the International Union for the Protection of New Varieties of Plants (UPOV), which require field trials to evaluate phenotypic traits. However, these methods can be subjective, require significant time and resources, and are influenced by environmental variability, which may result in classification inconsistencies (Cooke and Reeves, 2003; Wang et al., 2016; Yu and Chung, 2021). For instance, *Rubus* species often exhibit overlapping leaf traits due to hybridization, making it difficult to distinguish varieties reliably through naked-eye observation alone (Lu, 1983; Lawrence, 1986; Alice and Campbell, 1999; Sochor et al., 2018; Gao et al., 2023). Molecular markers, such as simple sequence repeats (SSRs) or single nucleotide polymorphisms (SNPs), offer greater precision but come with high costs, technical complexity, and the need for specialized laboratory equipment, limiting their accessibility for routine variety examinations (Liang et al., 2020; Jo et al., 2022). As a result, there is a pressing need for cost-effective, objective tools that can quantify subtle morphological variations while complementing existing protocols (Reynolds et al., 2020; Zhu et al., 2024).

Advancements in morphometric techniques have revolutionized plant phenotyping by enabling quantitative analysis of shape and structure (Evin et al., 2022; Spani et al., 2025). Geometric morphometrics (GM), in particular, transcends traditional phenotyping by capturing multidimensional data through landmark-based analysis (focusing on homologous anatomical points, such as leaf tips and vein intersections) and outline-based analysis (using Elliptic Fourier Descriptors [EFD] to model contours) (Manacorda and Asurmendi, 2018; Oso and Jayeola, 2021). Landmark analysis excels at detecting internal structural differences, like venation patterns, while EFD provides a robust representation of overall shape, even in complex or asymmetrical forms (Chitwood and Otoni, 2017; Klein et al., 2017). Hybrid approaches combining these methods have demonstrated superior discriminatory power in various taxa, including *Passiflora* species, where they quantified leaf shape variation and relationships between vasculature and blade contours (Chitwood and Otoni, 2017).

Despite extensive research on cane growth and reproductive differences between primocane (current-season vegetative canes) and floricane (previous-season fruiting canes) in *Rubus* (Thompson et al., 2007; Toshima et al., 2021; Medina et al., 2024), systematic comparisons of leaf shape variation between these ontogenetic stages remain scarce. Here, we evaluated the performance of geometric morphometric approaches by discriminating against 10 accessions of *R. crataegifolius* (comprising 8 varieties and 2 landraces), using leaf samples collected from both primocane and floricane stages. Specifically, we (1) applied landmark-based, outline-based (EFD), and hybrid analyses; (2) used principal component analysis (PCA) and linear discriminant analysis (LDA) to visualize separation and quantify discriminatory power; and (3) compared classification success rates across methods using confusion matrices and cross-validated accuracies.

## 2 Materials and methods

### 2.1 Plant material and image acquisition

Plant material consisted of ten accessions of *R. crataegifolius* Bunge maintained at the National Forest Seed Variety Center, Chungju, Republic of Korea (36.88°N, 127.98°E; approximately 150 m elevation). All accessions were grown under open-field conditions in a cultivar evaluation trial established in March 2024, with plants spaced at 1.0 m within and between rows. The original calcareous clay soil was amended with a 50 cm layer of decomposed granite soil (masato) mixed with sand to improve drainage. Standard horticultural management practices (annual pruning, fertilization, and irrigation) were applied uniformly across all accessions. These included four registered cultivars (**Table 1**) ‘Dongakheuk’ (DAHE), ‘Dongakhong’ (DAHO), ‘Geumdongcheon’ (GDC), and ‘Geumdongwang’ (GDW), four cultivars filed for plant variety protection ‘Dabok’ (DB), ‘Daesung’ (DS), ‘Goldmoon’ (GDM), and ‘Hwangeumboll’ (HGB), and two regional landraces collected from Gyeongnam (GN) and Jeonnam (JN) provinces. Of the eight registered or filed cultivars, seven (DAHE, DAHO, DB, DS, GDC, GDW, HGB) were developed by the Gyeongsangbuk-do Agricultural Research & Extension Services and one (GDM) was developed by the Jeonnam Forest Research Institute; full registration details, including application numbers, are summarized in **Table 1**. The two landraces were included to evaluate the capacity of morphometric methods to characterize previously unregistered accessions and to represent the range of wild-type morphological variation.

**Table 1:** Summary of the 10 *Rubus crataegifolius* accessions used in this study.

Abbreviation	Full name	Registration status	Breeder (origin)	Application no.
DAHE	Dongakheuk	Registered cultivar	Gyeongsangbuk-do ARES	2018-42
DAHO	Dongakhong	Registered cultivar	Gyeongsangbuk-do ARES	2019-33
DB	Dabok	Filed for PVP	Gyeongsangbuk-do ARES	2023-45
DS	Daesung	Filed for PVP	Gyeongsangbuk-do ARES	2023-46
GDC	Geumdongcheon	Registered cultivar	Gyeongsangbuk-do ARES	2019-32
GDM	Goldmoon	Filed for PVP	Jeonnam FRI	2023-32
GDW	Geumdongwang	Registered cultivar	Gyeongsangbuk-do ARES	2018-41
GN	Gyeongnam landrace	Regional landrace	Collected from Gyeongnam Province (wild)	—
HGB	Hwangeumboll	Filed for PVP	Gyeongsangbuk-do ARES	2023-44
JN	Jeonnam landrace	Regional landrace	Collected from Jeonnam Province (wild)	—

Gyeongsangbuk-do ARES, Gyeongsangbuk-do Agricultural Research & Extension Services; Jeonnam FRI, Jeonnam Forest Research Institute. All cultivars are maintained at the National Forest Seed Variety Center (NFSV), Chungju, Republic of Korea. “Breeder (Origin)” refers to the institution that developed and applied for cultivar registration with the Korea Forest Service; “Application No.” corresponds to the Korea Plant Variety Protection registry, where the prefix indicates the year of application. The two regional landraces (GN and JN) were collected from open-pollinated wild populations in Gyeongnam and Jeonnam provinces, respectively, and are maintained at NFSV alongside the registered cultivars.

Because primocane and floricane leaves reach full expansion at different phenological stages (Alvarado-Raya et al., 2007), sampling was timed to capture fully mature leaves of each cane type. Floricane leaves were collected in early May 2025, when leaves on overwintered canes were fully expanded, specifically prior to the onset of leaf senescence typically associated with fruit maturation after flowering, to ensure that significant fruit load had not yet altered. Primocane leaves were collected in early July 2025, when current-season vegetative canes had produced fully expanded mature foliage. To minimize ontogenetic variation within each type of cane, leaves were consistently taken from the 3rd to 4th fully expanded leaf below the apex of the cane. From each accession and cane type, 18 undamaged leaves were sampled per accession and cane type. For each accession, leaves were collected from six individual plants (three leaves per plant), following the sampling framework of the Korean Test Guidelines for Hawthornleaf Raspberry (Document No. Forest-26; Korea Forest Service, 2009), to incorporate within-accession biological variation, resulting in a total of 360 leaves (10 accessions × 2 cane types × 18 replicates).

Immediately after collection, each leaflet was placed adaxial surface down and scanned on the same day using a Sindoh Aficio 220F multifunction printer/scanner at 600 dpi. Images were saved as high-quality JPG files.

### 2.2 Geometric morphometric analysis

Analyses followed protocols adapted from Chitwood and Otoni (2017) and (Klein et al., 2017) for landmark- and outline-based approaches, integrating both to capture comprehensive shape variations, with specific adjustments for *Rubus* leaf venation and asymmetry.

**Figure 1:**
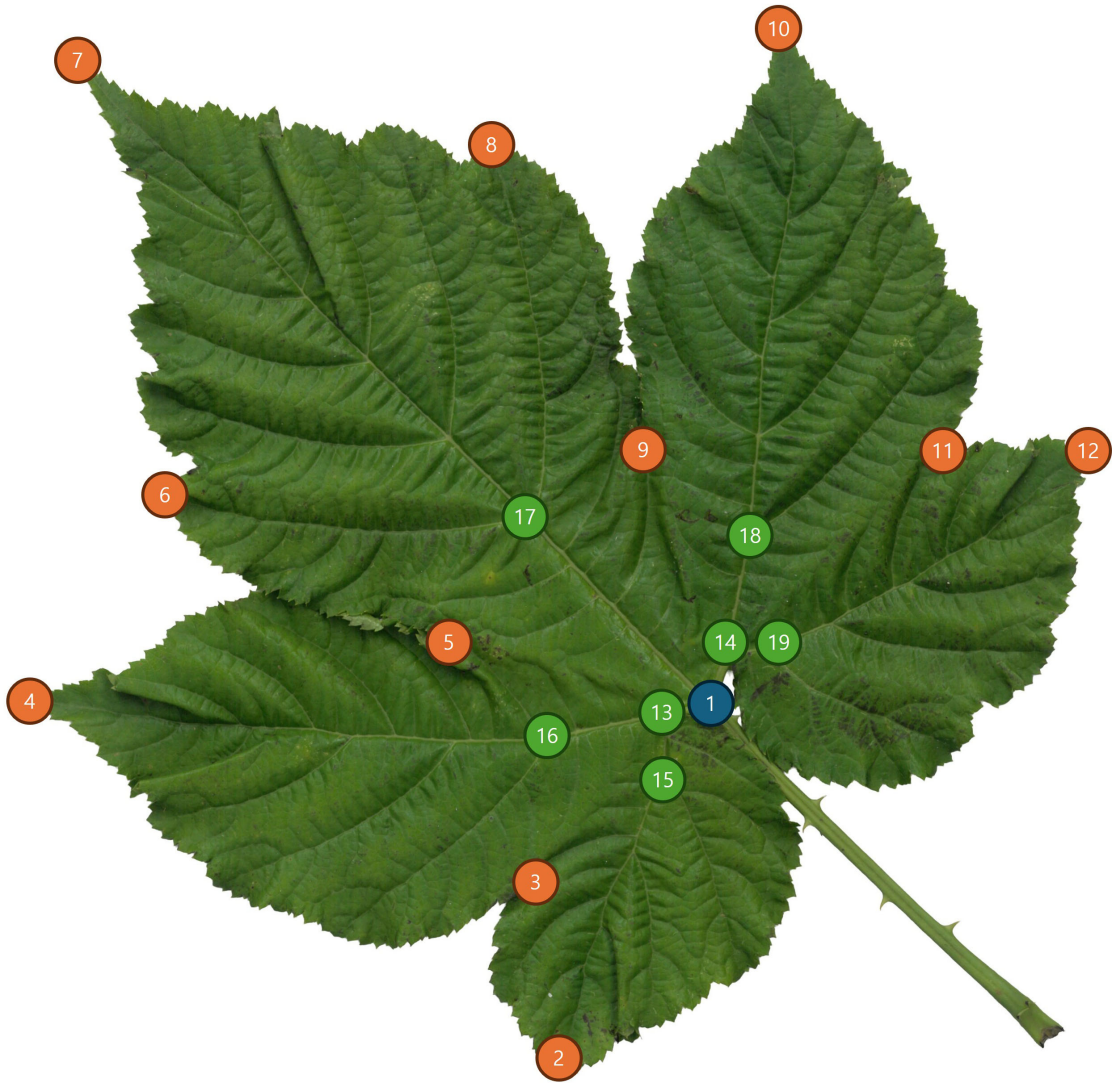
Example of landmark positions on a representative leaf of *Rubus crataegifolius*. Orange points (2–12) mark the leaf outline tips, green points (13–19) indicate vein junctions, and the blue point (1) denotes the petiole base.

Nineteen anatomical landmarks were digitized on each leaf image using ImageJ ver. 1.54g (Schneider et al., 2012). These captured *Rubus*-specific features: 12 outline points (leaflet tips, serration peaks, petiole base, overall base) and 7 vein points (primary/secondary vein junctions, intersections emphasizing palmate venation; see **Figure 1** for definitions and placement rationale). Coordinates (x, y) were exported as tab-delimited.txt files.

**Figure 2:**
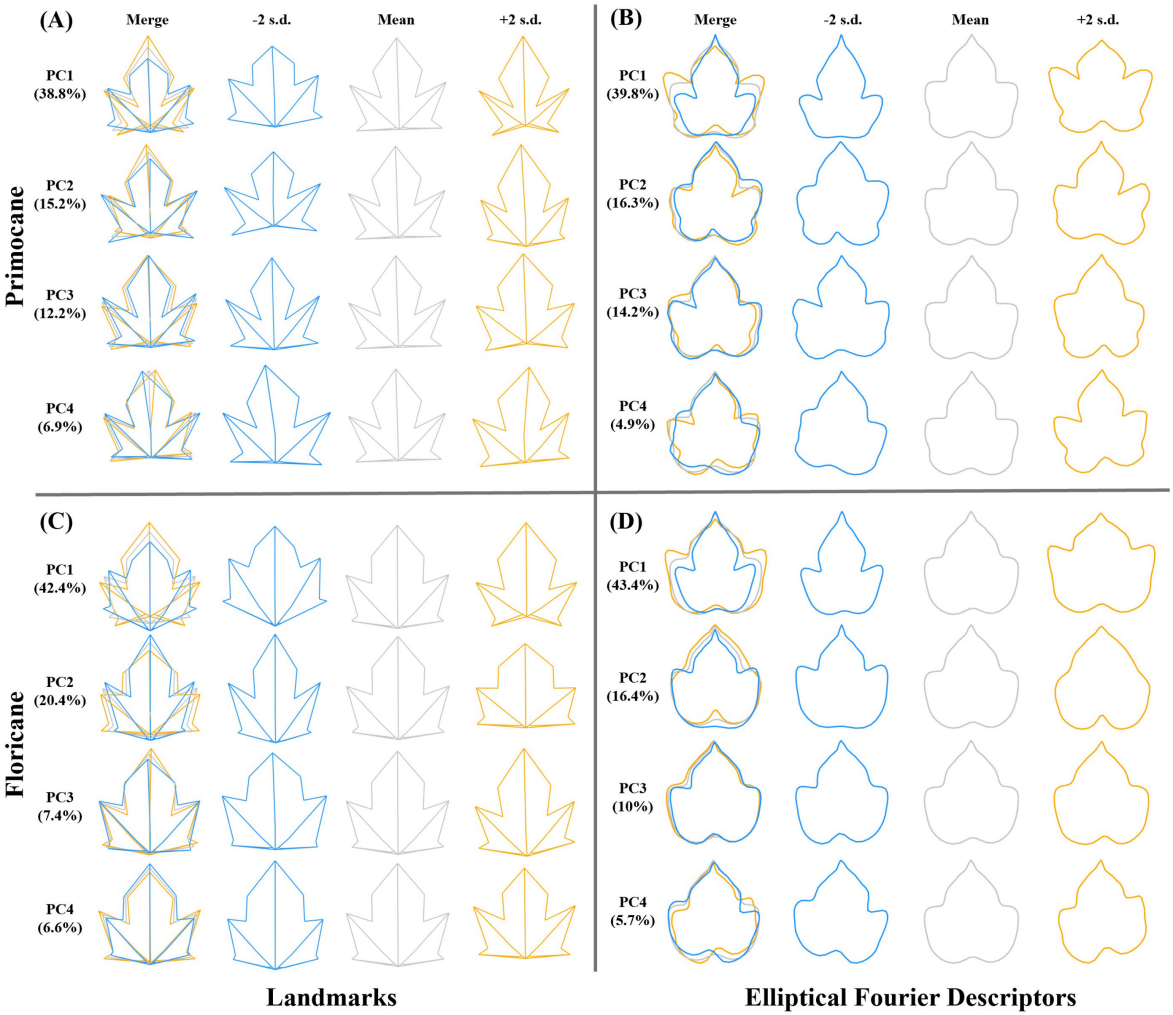
Eigenleaves illustrating morphological variations along the first four principal components (PCs) in leaf shapes. **(A)** Primocane leaves analyzed using landmarks and Generalized Procrustes Analysis (GPA); **(B)** Primocane leaves analyzed using outlines and Elliptic Fourier Descriptors (EFD); **(C)** Floricane leaves analyzed using landmarks and GPA; **(D)** Floricane leaves analyzed using outlines and EFD. In each panel, shapes are reconstructed at varying standard deviations from the mean (± 2 SD), with color gradients from blue (negative deviation) to yellow (positive deviation).

Generalized Procrustes Analysis (GPA) aligned configurations to remove non-shape effects (position, rotation, scale; reflection allowed for asymmetry) using procGPA in the shapes package ver. 1.2.7 (Dryden and Mardia, 2016) in R ver. 4.5.1 (R Core Team, 2025). Procrustes residuals were used for PCA to compute PC scores and variance explained. Shape deformations along principal axes were visualized as eigenleaf diagrams (i.e., shape deformation plots showing morphological variation along each PC axis) (± 2 s.d. from mean, with mean) using shapepca or custom ggplot2 plotting (Wickham, 2016) with connections for veins/leaflets (**Figures 2A, C**).

To assess intra-observer landmark digitizing error, a subset of 30 primocane leaves (3 per accession) was digitized three times independently by a single observer. Procrustes ANOVA partitioned total shape variance into among-variety (70.30%), within-variety among-individual (29.42%), and measurement error (0.27%) components. Repeatability was high (R = 0.996, F = 755.9, p = 0.001), with per-variety values ranging from 0.981 (JN) to 0.995 (HGB), confirming that digitizing error was negligible relative to biological variation.

Scanned images were converted to binary format to isolate leaf contours and Elliptic Fourier Descriptors (EFDs) were then computed to quantify outline shapes using SHAPE ver. 1.3 (Iwata and Ukai, 2002), generating chain-code representations (.chc files) of the biological contours. Standardized EFD coefficients were imported into the Momocs package ver. 1.4.1 (Bonhomme et al., 2014) in R, where the first 20 harmonics (A, B, C, D coefficients) were extracted and normalized for rotation, size, and starting point, ensuring 99% of shape variance was captured (as determined by cumulative power analysis; **Supplementary Figure 1**). Harmonic contributions to overall shape were assessed with the hcontrib function, mean shapes were calculated using meanShapes, and principal component analysis was performed via the pca function. Contour variations were illustrated through eigenleaf plots generated by PC.contrib (± 2 s.d., mean; **Figures 2B, D**).

A hybrid dataset concatenated Procrustes residuals (landmarks) and EFD coefficients (outlines) for integrated analyses, enabling evaluation of vasculature-blade relationships. No pre-scaling was applied to the hybrid dataset, as LDA operates on the within-group covariance structure and is therefore scale-invariant; this was confirmed empirically by comparing results with and without z-score standardization, which produced identical classification accuracies.

### 2.3 Multivariate and statistical analyses

All statistical procedures were conducted in R ver. 4.5.1 (R Core Team, 2025). Separate analyses were performed for primocane and floricane datasets to account for ontogenetic differences, with combined analyses where appropriate.

To classify varieties and cane types (primocane vs. floricane), LDA was implemented using the lda function in the MASS package ver. 7.3.65 (Venables and Ripley, 2002), with leave-one-out cross-validation (LOOCV) to estimate classification accuracy. Because GPA-aligned Procrustes coordinates are structurally rank-deficient (effective dimensionality = 2k − 4 = 34 for k = 19 landmarks in 2D; Dryden and Mardia, 2016), direct use of raw coordinates as LDA predictors introduces multicollinearity. To address this, PCA was first applied to Procrustes residuals, and the resulting 34 orthogonal principal component scores were used as predictors in LDA for landmark-based and hybrid models, thereby retaining all shape information from 19 landmarks while eliminating multicollinearity. 3D scatter plots of LD scores were generated using ggplot2 and plotly (Plotly Technologies Inc, 2015).

Predictions generated confusion matrices (base table(); proportions via prop.table()), where confusion matrices were derived from these LD score-based classifications to visualize actual vs. predicted identities as heatmaps with ggplot2, highlighting diagonal elements with percentages of correct classifications.Inter-variety morphological relationships were quantified by computing Pearson correlation coefficients (r) on variety-averaged principal component scores (n = 34 PCs per variety, derived from the 34 effective shape dimensions of 19 Procrustes-aligned landmarks). Statistical significance of each pairwise correlation was assessed using cor.test with df = 32. Heatmaps were produced with the corrplot.mixed function from the corrplot package (Wei and Simko, 2024), employing diverging color palettes (e.g., RdYlBu) and mixed visualizations (numbers in the lower triangle, circles in the upper) for enhanced interpretability.To evaluate congruence between landmark- and outline-based datasets, Euclidean distance matrices were computed for each and compared using the mantel function in the vegan package version 2.6-2 (Oksanen et al., 2025), with significance assessed via 999 permutations.

For evidentiary assessment relevant to regulatory DUS contexts, three complementary analyses were conducted on the LOOCV outputs: (i) exact 95% binomial confidence intervals for overall and per-variety classification accuracies (binom.test); (ii) two label-permutation tests (999 shuffles each, with full pipeline re-fitting per shuffle): a leaf-level permutation that randomly reassigns the variety label of each of the 180 individual leaves, and a more conservative plant-level permutation that preserves the nested 6 plants × 3 leaves design (the three leaves of each plant are kept together and assigned to the same shuffled variety, with the per-variety plant count held fixed at six (plant identity within each accession was inferred from the leaf numbering, with consecutively numbered triples treated as belonging to the same plant); and (iii) pairwise within-pair classification accuracy derived from the 10-class confusion matrix as correct/(correct + cross-confused), with binomial 95% CIs. As a complementary (hypothesis-testing-framework) assessment, pairwise Mahalanobis D² distances between variety centroids on the retained PCs were converted to Cohen’s f² and used to compute achieved statistical power at α = 0.05 via pwr.f2.test from the pwr package ver. 1.3-0 (Champely, 2020); we note that this F-test framework was designed for hypothesis-testing rather than classification problems and is reported here only as a conservative supplementary benchmark.

## 3 Results

### 3.1 Leaf shape variation and inter-variety relationships

PCA of leaf shapes from all cultivars, using landmarks and EFDs, showed that the first four PCs collectively explained more than 70% of total morphological variance in both methods (**Figure 2**). Notably, PC1 accounted for a lower proportion of variance in primocane leaves compared to floricane leaves, with a consistent difference of 3.6 percentage points in both landmark (38.8% vs. 42.4%) and EFD (39.8% vs. 43.4%) analyses, indicating that global shape diversity in primocane leaves is less concentrated along a single dominant axis and more dispersed across finer-scale components.

Across both landmark and outline analyses, PC1 primarily captured variation in leaf base morphology (particularly the angle of the lowermost veins) and blade length-to-width ratio. PC2 reflected the depth of the upper lateral sinus, PC3 described the slope or spreading angle of the upper lobes, and PC4 was associated with symmetry between the leaf base and blade. These patterns suggest that, while primocane and floricane leaves differ in the distribution of variance, the major axes of shape variation consistently relate to basal structure, lobe configuration, and overall symmetry in leaves of *R. crataegifolius*.

**Figure 3:**
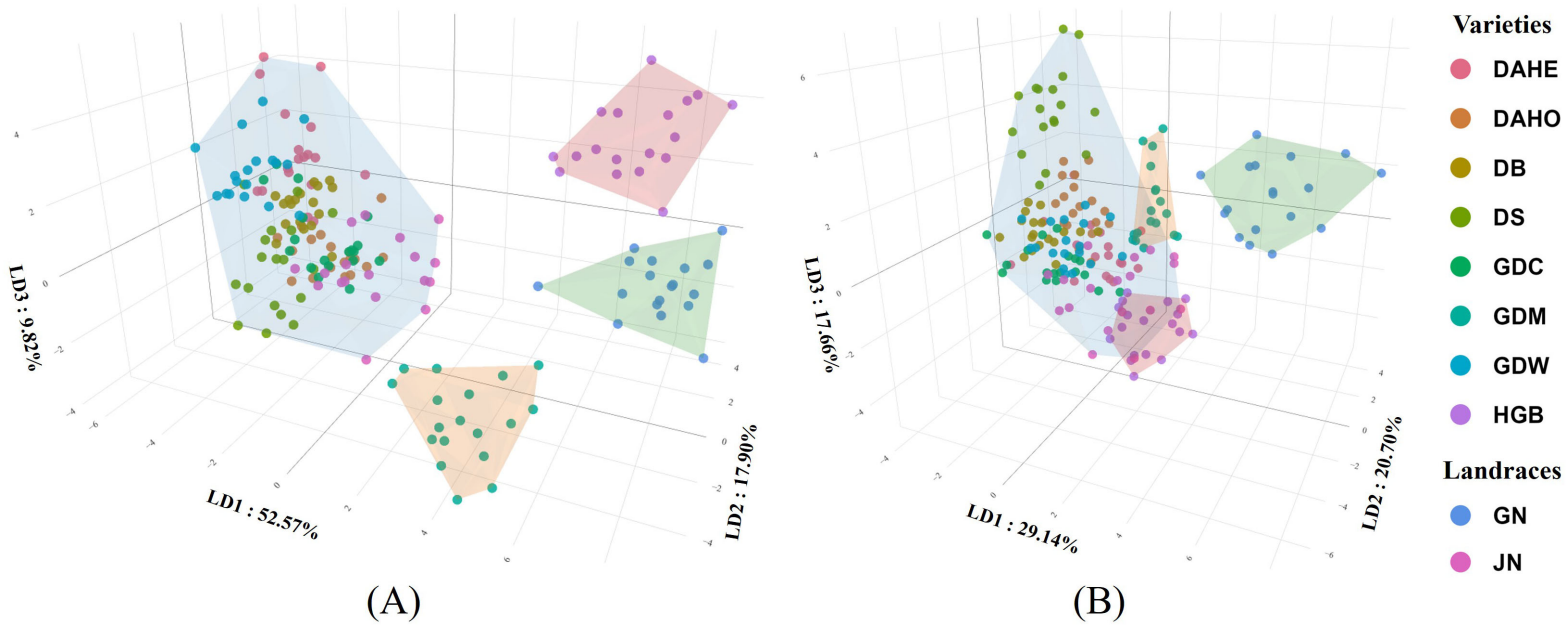
Morphometric clustering based on PCA-LDA of 19 Procrustes-aligned landmarks (34 orthogonal PC scores) for primocane **(A)** and floricane **(B)** leaves of *Rubus crataegifolius*. The 3D plots show LD1, LD2, and LD3 scores. Varieties (DAHE, DAHO, DB, DS, GDC, GDM, GDW, HGB) and landraces (GN, JN) are color-coded, with convex hulls highlighting four morphological groups. Variance proportions are labeled on axes, reflecting the discriminatory power of the analysis.

**Figure 4:**
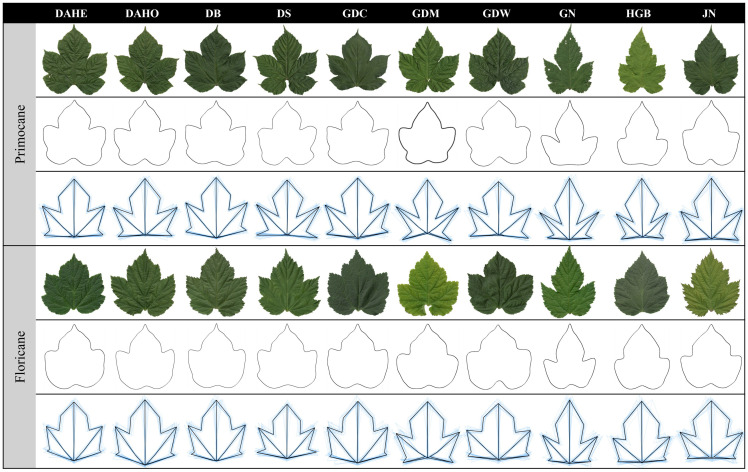
Representative leaf shapes for primocane (top) and floricane (bottom) stages across different accessions (DAHE, DAHO, DB, DS, GDC, GDM, GDW, GN, HGB, JN). For each variety and stage, shown are scanned images of actual leaves (top row per section), outlines derived from Elliptic Fourier Descriptors (EFD) analysis (middle row per section), and landmark configurations from Generalized Procrustes Analysis (GPA) (bottom row per section).

The clearest morphological separation among the 10 *R. crataegifolius* varieties and landraces was achieved using PCA-LDA on Procrustes-aligned landmark coordinates (19 landmarks, 34 PCs). This approach produced well-defined clustering, especially in the primocane ordination (**Figure 3A**; LD1: 52.57%, LD2: 17.90%, LD3: 9.82%). These separations are visually corroborated by the mean primocane leaf shapes and superimposed outlines (**Figure 4**), where differences in basal shape, vein angles, lobe elongation, and margin dissection drive the group distinctions.

In the primocane LDA plot, the largest cluster (left side, negative LD1 scores; **Figure 3A**) grouped DAHE, DAHO, DB, DS, GDC, GDW, and JN. These accessions shared broader, less deeply lobed, and more rounded leaf outlines, suggesting a common morphotype, although gradient variation is still observed among varieties by LD scores. DS was positioned adjacent to the main group, with partial overlap in the LD1-LD3 plane but separation along LD2 (DS mean LD2 = -3.67 vs. Group 1 mean = +0.63); despite visual proximity, DS maintained 88.9% LOOCV classification accuracy, indicating effective discrimination across higher-order discriminant axes (see also **Supplementary Figure 2** for 2D pairwise LD plots). The remaining three varieties formed distinct, well-separated clusters: GDM showed wider basal angles with downward-oriented leaf base tips; GN displayed elongated leaves with distally extended lobes; HGB featured elongated leaves with downward-oriented leaf base tips. The corresponding floricane LDA plot exhibited a similar but less compact clustering pattern (**Figure 3B**), consistent with the lower overall LOOCV accuracy for floricane leaves reported below.

**Figure 5:**
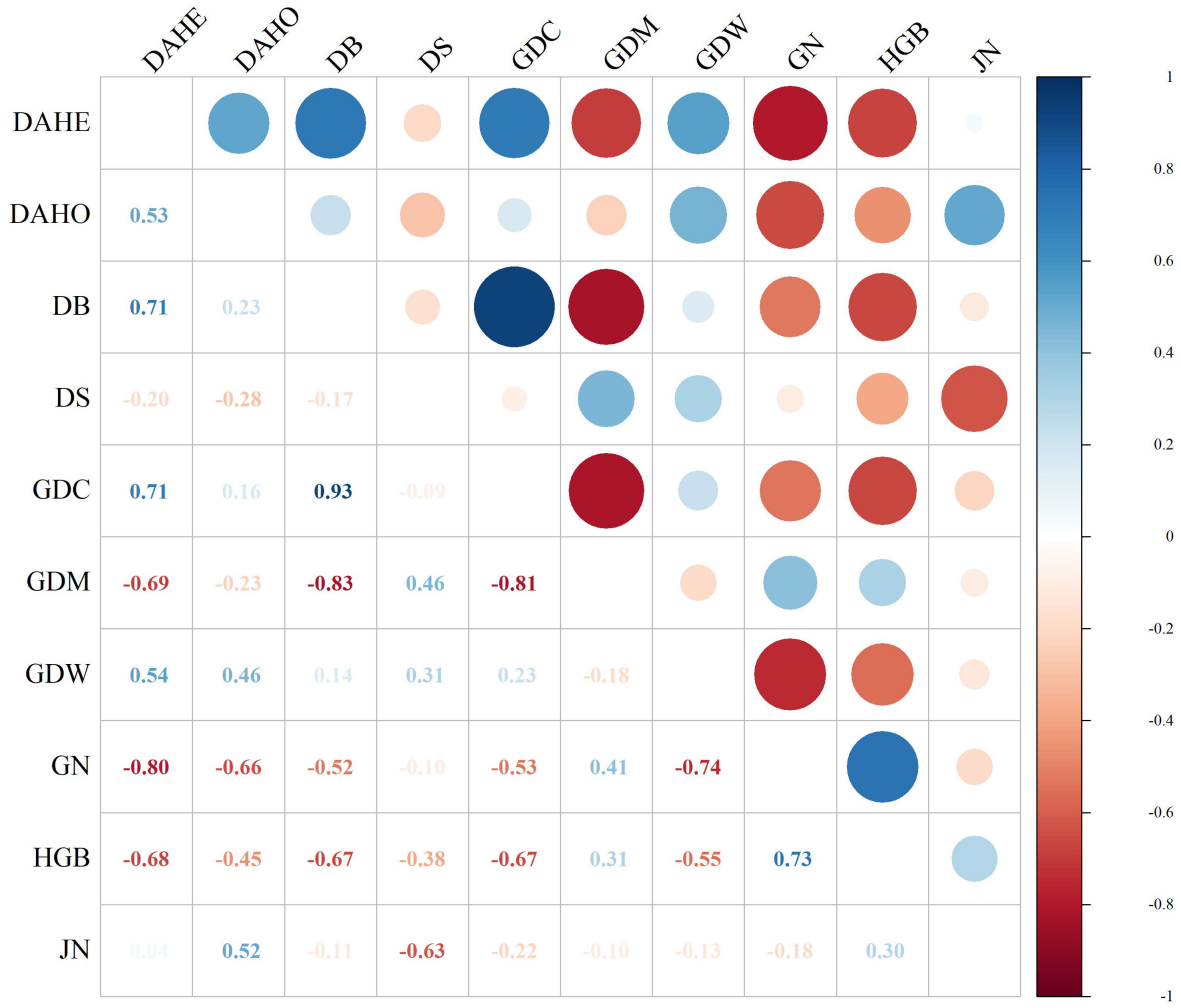
Correlation matrix between varieties of *Rubus crataegifolius* based on variety-averaged principal component scores (n = 34 effective PCs derived from 19 Procrustes-aligned landmarks) for primocane leaves. Pearson correlation coefficients are displayed as numbers in the lower triangle and circles in the upper triangle. Of the 45 pairwise correlations, 25 were statistically significant (p < 0.05).

These morphological distinctions are further supported by pairwise correlation analysis based on variety-averaged PC scores (n = 34; **Figure 5**). Within the broad morphotype group, DB and GDC showed the strongest positive correlation (r = 0.93, p < 0.001), and DAHE was also positively correlated with DB (r = 0.71, p < 0.001) and GDC (r = 0.71, p < 0.001). In contrast, GN showed strong negative correlations with DAHE (r = -0.80, p < 0.001), GDW (r = -0.74, p < 0.001), and DAHO (r = -0.66, p < 0.001), confirming its morphological distinctiveness. GDM was negatively correlated with DB (r = -0.83, p < 0.001) and GDC (r = -0.81, p < 0.001), consistent with its separation in the LDA ordination. GN and HGB maintained a positive correlation (r = 0.73, p < 0.001), reflecting their shared deep-sinused leaf architecture. Of the 45 pairwise correlations, 25 were statistically significant (p < 0.05), with 23 significant at p < 0.01.

### 3.2 Predictive performance and model comparison across datasets and cane types

**Figure 6:**
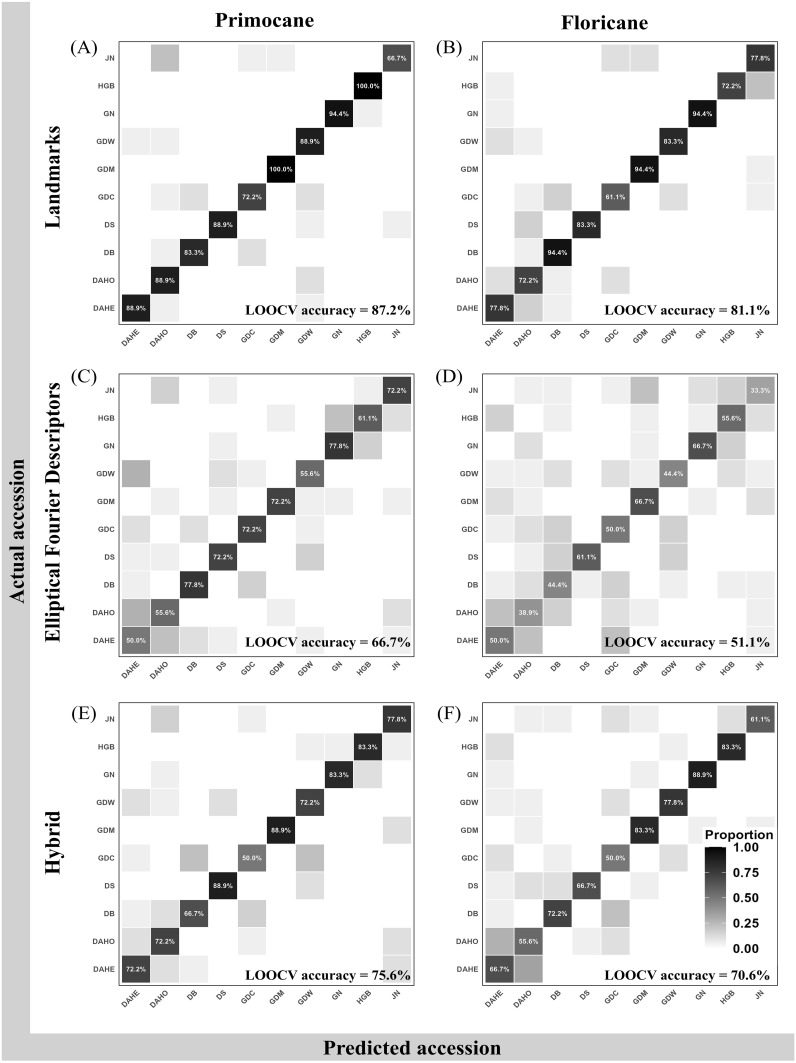
Confusion matrices for variety classification of *Rubus crataegifolius* using LDA on landmark (PCA-LDA, 19 landmarks), outline (EFD), and hybrid (PCA-LDA + EFD) data. Diagonal values represent classification accuracies (%), with rows as actual varieties and columns as predicted accessions (sorted alphabetically: DAHE, DAHO, DB, DS, GDC, GDM, GDW, GN, HGB, JN). Leave-one-out cross-validation (LOOCV) accuracy is presented in the bottom right of each matrix. **(A)** Primocane leaves using landmark data; **(B)** Floricane leaves using landmark data; **(C)** Primocane leaves using EFD data; **(D)** Floricane leaves using EFD data; **(E)** Primocane leaves using hybrid data; **(F)** Floricane leaves using hybrid data.

We evaluated the discriminatory power of three morphometric approaches (landmarks only, EFDs only, and a combined landmark + EFD dataset) using LDA with LOOCV for variety classification in *R. crataegifolius* leaves (**Figure 6**). Analyses were conducted separately for primocane and floricane leaves to assess the influence of cane type on morphometric resolution.

For primocane leaves, the landmarks-only model achieved the highest overall classification accuracy (87.2%; **Figure 6A**), surpassing the combined hybrid dataset (75.6%; **Figure 6E**) and the EFDs-only approach (66.7%; **Figure 6C**). Similarly, for floricane leaves, the landmarks-only model performed best (81.1%; **Figure 6B**), followed by the combined hybrid dataset (70.6%; **Figure 6F**) and EFDs-only (51.1%; **Figure 6D**). Primocane accuracies exceeded floricane across all methods, reflecting greater uniformity. Exact 95% binomial confidence intervals confirmed both overall accuracies as highly precise (primocane [81.4%, 91.7%]; floricane [74.6%, 86.5%]), and label-permutation tests (999 shuffles each) demonstrated that these accuracies are not attributable to chance (P < 0.001) under both leaf-level and a more conservative plant-level scheme that preserves the nested 6 plants × 3 leaves design (null maxima 28.9–29.4% vs. 20.6% for leaf-level).

Examination of the confusion matrices (**Figure 6**) revealed distinct misclassification patterns across methods and cane types. In the primocane landmark analysis (87.2% overall), GDM and HGB achieved perfect classification (100%), while JN showed the lowest accuracy (66.7%), with misclassifications primarily toward DAHO (22.2%). GDC was most frequently confused with DB and GDW (11.1% each), consistent with their morphological proximity in the LDA ordination. In the floricane landmark analysis (81.1%), DB, GDM, and GN each achieved 94.4% accuracy. GDC showed the lowest floricane accuracy (61.1%), declining from 72.2% in the primocane analysis, while DAHO and HGB followed at 72.2% each. GN and HGB consistently maintained high primocane landmark classification rates (94.4-100%), reflecting their morphologically distinct deep-sinused leaf architecture.

Pairwise confusability rates derived from the 10-class confusion matrix showed that 32 of 45 primocane variety pairs (71%) and 31 of 45 floricane pairs (69%) exhibited zero direct A↔B cross-confusion, and the lower bound of the binomial 95% CI exceeded 80% for 42 (primocane) and 38 (floricane) of the 45 pairs; the most-confused pairs were DAHO–JN and DB–GDC for primocane (88.9% retention each, 95% CI [73.9%, 96.9%]) and DAHE–DAHO for floricane (86.1%, 95% CI [70.5%, 95.3%]).

Mantel tests on Euclidean distance matrices showed moderate congruence between landmarks and EFDs for primocanes (r = 0.5161, p = 0.001) but weaker for floricanes (r = 0.2876, p = 0.001), indicating differential impacts of plasticity on landmarks versus EFDs.

## 4 Discussion

### 4.1 Methodological strengths and developmental influences on morphometric performance in primocane and floricane leaves

The landmarks-only approach demonstrated the most consistent superiority across both cane types, emphasizing the reliability of vascular landmarks in detecting key diagnostic traits like vein angles, branching patterns, sinus positions, and lobe placements. These internal features maintain stability amid developmental plasticity or positional differences along the shoot. By contrast, EFD-based outline descriptors proved generally less effective, likely because they are less attuned to subtle internal elements such as overlapping or nested lobes—details that homologous landmarks on vascular and margin structures resolve more effectively in *R. crataegifolius*. This finding is consistent with Chitwood and Otoni (2017), who reported that landmark analyses captured vascular architecture details that outline-based EFD alone could not resolve in *Passiflora* leaves. Similarly, Manacorda and Asurmendi (2018) demonstrated that geometric morphometrics outperformed human visual assessment in detecting subtle shape differences in *Arabidopsis*, further supporting the sensitivity of landmark-based approaches. That said, EFDs-only or combined methods occasionally surpassed landmarks for particular accessions (e.g., higher accuracy for primocane JN with EFDs and the hybrid dataset), suggesting that contour data can enhance vascular information and boost discrimination in cases where outline variations are prominent.

The superior classification accuracy of primocanes relative to floricanes may be associated with differences in developmental timing and environmental conditions, although this interpretation remains a hypothesis that requires direct experimental validation. Collected in July, primocanes may have benefited from relatively stable midsummer growing conditions compared to the more variable spring environment experienced by floricanes. Studies on *Rubus idaeus* have reported higher and more stable photosynthetic rates in primocanes (Alvarado-Raya et al., 2007), though whether this pattern extends to *R. crataegifolius* remains to be confirmed. In contrast, floricanes were subjected to vernal environmental fluctuations and the physiological demands of reproductive transition, which likely heightened phenotypic noise. These results suggest that primocane foliage serves as a more reliable diagnostic marker for geometric morphometric characterization due to its reduced ontogenetic and environmental variability.

Mantel test results further support this, with stronger method alignment in primocanes reflecting their stability and enabling consistent inter-variety distance rankings. The weaker congruence in floricanes points to heightened plasticity, which may impact vascular (landmarks) and contour (EFDs) traits differently. Notably, the inclusion of all 19 landmarks, including vein junction points, improved floricane classification from 51.1% (i.e., EFD only) to 70.6% (i.e., hybrid), suggesting that vein architecture provides additional discriminatory information in floricane leaves where outline-level differences are reduced by environmental plasticity. Overall, these findings reveal partial methodological overlap while underscoring their complementary nature, accounting for the sporadic benefits seen in hybrid analyses.

To contextualize our classification accuracy, we compared our results with previous geometric morphometric studies on plant leaves. At the interspecific level, Klein et al. (2017) achieved 98.4% classification accuracy for two *Vitis* species using landmark and EFD analyses, but genotype-level discrimination within species dropped to only 66% using resubstitution. Chitwood and Otoni (2017) reported LOOCV accuracies of 83.7-96.6% across classes of 40 *Passiflora* species using combined landmark and EFD data. Our 87.2% LOOCV accuracy for 10 *R. crataegifolius* cultivars using landmark analysis of primocane leaves compares favorably with these benchmarks, particularly given that intraspecific cultivar discrimination is a substantially harder task than interspecific classification. A previous morphological study on *R. crataegifolius* leaf variation (Cho et al., 2009) employed traditional linear measurements across 11 populations, identifying significant inter-population variation but without quantitative classification accuracy metrics. Our study thus represents the first application of geometric morphometrics to *R. crataegifolius* cultivar identification, demonstrating that landmark-based approaches can achieve cultivar-level discrimination comparable to or exceeding results reported for other crop species.

### 4.2 Applicability of morphometric techniques to DUS testing

The robust discriminatory power observed in our morphometric analyses, particularly the 87.2% classification accuracy for primocane leaves using landmark-based methods, highlights the potential of geometric morphometrics as a complementary tool in Distinctness, Uniformity, and Stability (DUS) testing for *R. crataegifolius* varieties. Traditional DUS protocols rely on subjective visual assessments and basic measurements over multi-year field trials, leading to inconsistencies due to observer bias and phenotypic plasticity. For example, the current Korean Test Guidelines for *R. crataegifolius* (Document No. Forest-26; Korea Forest Service, 2009) assess leaf traits through a combination of visual scoring (e.g., leaf apex, leaf margin, leaf blade shape) and caliper-based measurement (e.g., leaf blade length, width, and length-to-width ratio), with all results reported as 1–9 ordinal classes. This framework is susceptible to inter-observer variability in the visually scored traits and reduces the resolution of measured traits through ordinal categorization. In contrast, morphometrics offers an objective, quantitative approach to capture multidimensional variations like vein angles and lobe symmetries, with cost-effective tools such as ImageJ and R packages enabling high-resolution analysis from minimal samples. Our findings suggest that these techniques are compatible with UPOV guidelines, offering high accuracy as a preliminary screening tool to optimize resources in *Rubus* breeding, especially where hybridization complicates variety distinctions.

Despite strengths, morphometrics requires standardized protocols to minimize imaging noise and integration with field evaluations for whole-plant traits like fruit yield; moreover, suitable plant organs—such as leaves in *Rubus*—may differ by species, potentially focusing on flowers, fruits, or roots in other crops. Future refinements, including machine learning for automation and expanded datasets, could test applicability beyond *Rubus* to diverse varieties like grains or vegetables.

Notably, the hybridization challenges discussed here primarily refer to spontaneous interspecific hybridization between *R. crataegifolius* and closely related sympatric *Rubus* species, rather than controlled intraspecific crosses within breeding programs.

In summary, morphometric analysis occupies a complementary position between traditional visual assessment and molecular marker analysis. While traditional DUS methods rely on subjective categorical scoring of limited traits (UPOV, 2023), and molecular approaches such as SSR or SNP genotyping require specialized laboratory infrastructure and expertise (Liang et al., 2020; Jo et al., 2022), geometric morphometrics offers objective, quantitative phenotypic data obtainable from standard imaging equipment. Morphometric profiles could serve as a preliminary screening tool to prioritize accessions for more costly molecular verification, or to supplement molecular distinctness data with phenotypic evidence in variety registration applications, thereby accelerating variety protection and supporting sustainable horticulture.

Furthermore, our findings show that morphologically similar varieties can be effectively distinguished using vegetative organs (leaves) alone, enabling variety verification at an early growth stage without requiring flowering or fruiting. This offers practical advantages for resolving seed distribution disputes and supports the integrity of the seed market.

## 5 Limitations and future directions

Several limitations should be acknowledged. (i) Data comes from a single growing season (2025) and one experimental site (NFSV, Chungju); multi-year and multi-site validation is required for broader generalization (Cho et al., 2009). (ii) The current n = 18 leaves per accession (six plants × three leaves; following the Korean Test Guidelines for Hawthornleaf Raspberry, Document No. Forest-26; Korea Forest Service, 2009) yields a ±5% half-width 95% CI on overall primocane accuracy but ±5.7% for floricane (n ≈ 24 needed for matching precision). A supplementary F-test power benchmark (pwr.f2.test; Champely, 2020) on the 34-dimensional feature space — designed for hypothesis-testing rather than classification — indicates n ≈ 21 leaves per accession as the minimum for ≥ 0.80 power on every pairwise comparison. Expanded sampling toward UPOV TG/43 standards for *R. idaeus* (n = 30, i.e., 10 plants × 3 leaves; UPOV, 2023) is therefore advisable for regulatory DUS deployment, particularly to tighten per-variety and per-pair CIs and better characterize within-accession variation. (iii) All digitization was performed by a single operator; inter-observer reproducibility should be evaluated as a priority in future protocol-standardization studies, with a minimum of three independent observers digitizing a shared subset of leaves. (iv) Only terminal leaflets were analyzed; lateral leaflets or whole compound leaf architecture may provide additional discriminatory information. Future work should address these multi-year, multi-site, and protocol-validation directions, alongside expansion to more registered varieties and breeding lines and comparison with traditional caliper-based DUS measurements.

## Data Availability

The raw data supporting the conclusions of this article will be made available by the authors, without undue reservation.
