# Supplementary material for: Application of geometric morphometrics for variety identification in Rubus crataegifolius: a comparison of primocane and floricane leaf morphology
Source: Front Plant Sci. 2026 Jul 14;17:1799767. doi: 10.3389/fpls.2026.1799767 (PMC13408251; doi:10.3389/fpls.2026.1799767)
Supplement: Supplementary file 1 [file SupplementaryFile1.docx]

**Supplementary Material**

Application of Geometric Morphometrics for Variety Identification in *Rubus crataegifolius*: A Comparison of Primocane and Floricane Leaf Morphology

Seol-Jong Kim, Yoon-Young Kim, Je Min Park, and Soon-Ho Kwon*

National Forest Seed Variety Center (NFSV), Chungju, Chungcheongbuk-do, Republic of Korea

**Supplementary Figure S1.**


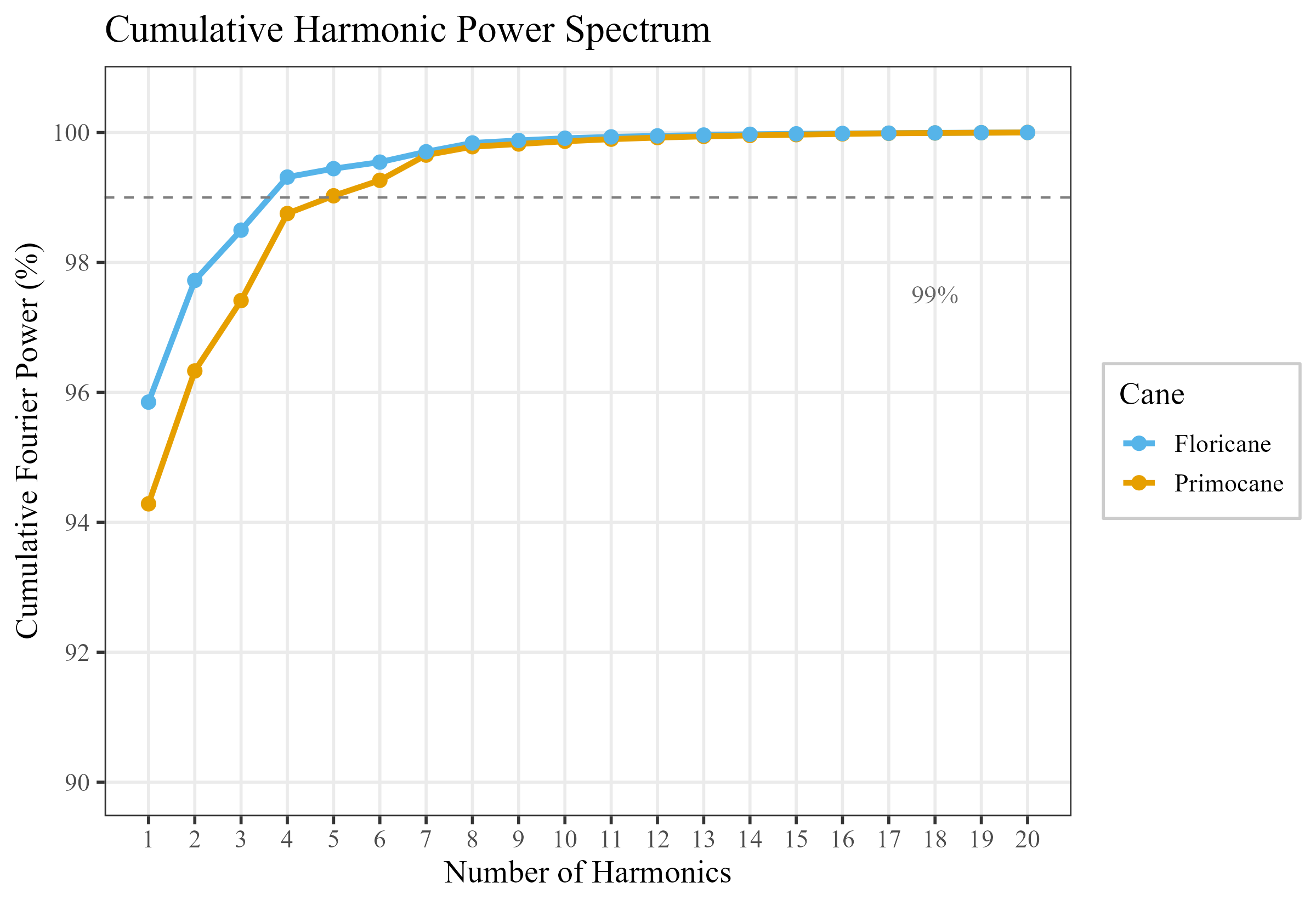


**Supplementary Figure S1.** Cumulative Fourier power spectrum for Elliptic Fourier Descriptor (EFD) analysis of *Rubus crataegifolius* leaf contours. The x-axis shows the number of harmonics (1-20), and the y-axis shows the cumulative proportion of shape variance captured, expressed as a percentage. Curves are shown separately for primocane (orange) and floricane (blue) leaves. The horizontal dashed line marks the 99% threshold, which was reached by approximately the 4th harmonic for floricane leaves and the 5th harmonic for primocane leaves. The first 20 harmonics were retained for subsequent analyses to ensure >99% of shape variance was preserved.

**Supplementary Figure S2.**


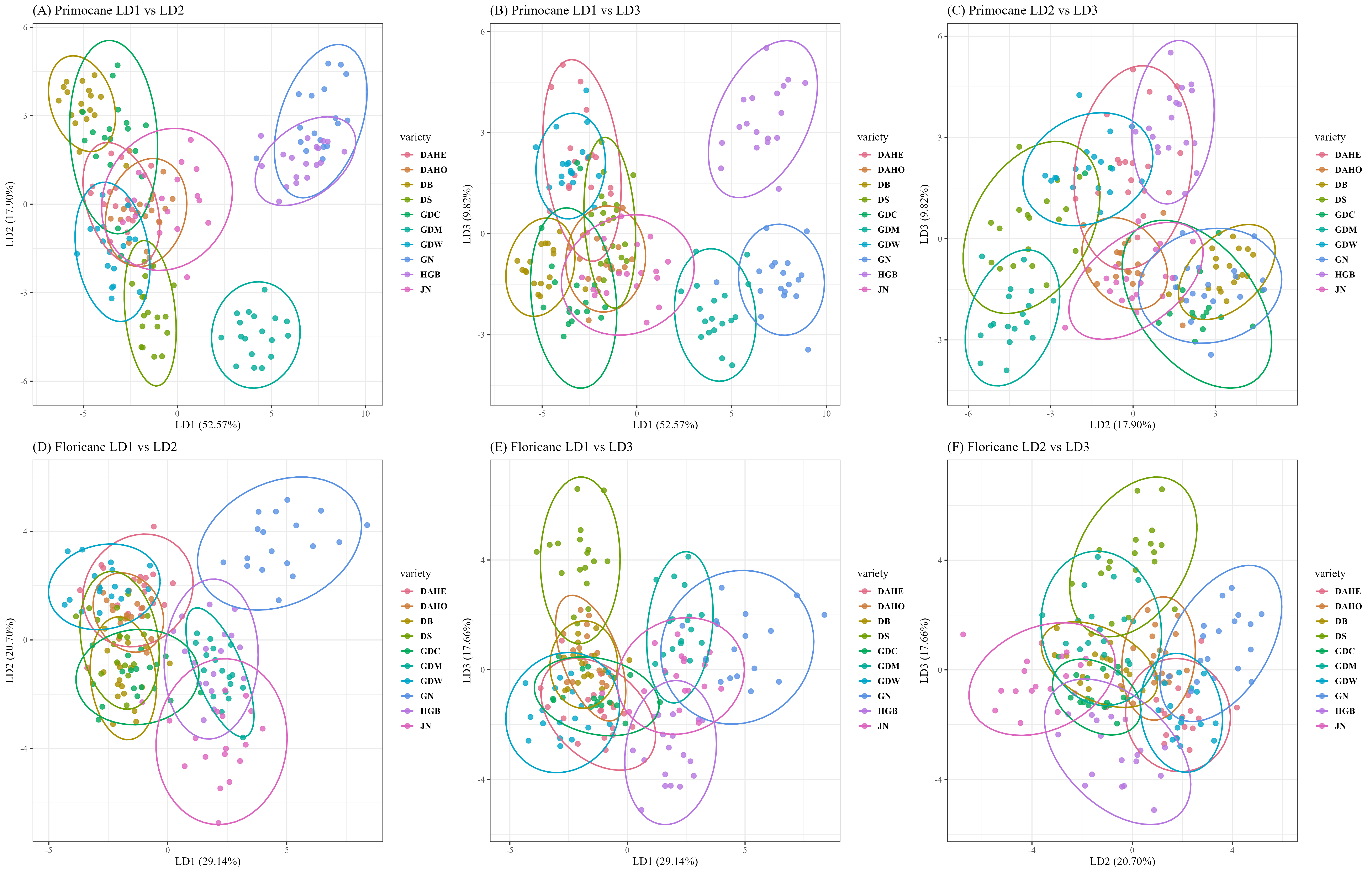


**Supplementary Figure S2.** Two-dimensional pairwise linear discriminant (LD) score plots from PCA-LDA based on 19 Procrustes-aligned landmark coordinates for primocane (top row: A-C) and floricane (bottom row: D-F) leaves of *Rubus crataegifolius*. Each panel shows a different combination of the first three LD axes: (A, D) LD1 vs. LD2; (B, E) LD1 vs. LD3; (C, F) LD2 vs. LD3. Points represent individual leaves; colors denote varieties (DAHE, DAHO, DB, DS, GDC, GDM, GDW, HGB) and landraces (GN, JN). Ellipses show 95% confidence regions for each accession. Percentages on axis labels indicate the proportion of between-group variance explained by each LD axis. These 2D projections complement the 3D LD plots shown in Figure 3 and illustrate discrimination along higher-order discriminant axes, supporting the classification of DS (88.9% LOOCV accuracy) despite its visual proximity to the large basal group in the primary LD1-LD2 plane.
